# Using the Lives Saved Tool to aid country planning in meeting mortality targets: a case study from Mali

**DOI:** 10.1186/s12889-017-4749-y

**Published:** 2017-11-07

**Authors:** Youssouf Keita, Hamadoun Sangho, Timothy Roberton, Emilia Vignola, Mariam Traoré, Melinda Munos, for the Mali NEP Working Group

**Affiliations:** 1 Institute for International Programs, Johns Hopkins University Bloomberg School of Public Health, Bamako, Mali; 2 Centre de Recherche, d’Etudes et de Documentation pour la Survie de l’Enfant (CREDOS), Bamako, Mali; 3 Institute for International Programs, Johns Hopkins University Bloomberg School of Public Health, Baltimore, MD USA

**Keywords:** Mali, National evaluation platform, Lives saved tool

## Abstract

**Background:**

Mali is one of four countries implementing a National Evaluation Platform (NEP) to build local capacity to answer evaluation questions for maternal, newborn, child health and nutrition (MNCH&N). In 2014-15, NEP-Mali addressed questions about the potential impact of Mali’s MNCH&N plans and strategies, and identified priority interventions to achieve targeted mortality reductions.

**Methods:**

The NEP-Mali team modeled the potential impact of three intervention packages in the Lives Saved Tool (LiST) from 2014 to 2023. One projection included the interventions and targets from Mali’s ten-year health strategy (PDDSS) for 2014-2023, and two others modeled intervention packages that included scale up of antenatal, intrapartum, and curative interventions, as well as reductions in stunting and wasting. We modeled the change in maternal, newborn and under-five mortality rates under these three projections, as well as the number of lives saved, overall and by intervention.

**Results:**

If Mali were to achieve the MNCH&N coverage targets from its health strategy, under-5 mortality would be reduced from 121 per 1000 live births to 93 per 1000, far from the target of 69 deaths per 1000. Projections 1 and 2 produced estimated mortality reductions from 121 deaths per 1000 to 70 and 68 deaths per 1000, respectively. With respect to neonatal mortality, the mortality rate would be reduced from 39 to 32 deaths per 1000 live births under the current health strategy, and to 25 per 1000 under projections 1 and 2.

**Conclusions:**

This study revealed that achieving the coverage targets for the MNCH&N interventions in the 2014-23 PDDSS would likely not allow Mali to achieve its mortality targets. The NEP-Mali team was able to identify two packages of MNCH&N interventions (and targets) that achieved under-5 and neonatal mortality rates at, or very near, the PDDSS targets. The Malian Ministry of Health and Public Hygiene is using these results to revise its plans and strategies.

**Electronic supplementary material:**

The online version of this article (10.1186/s12889-017-4749-y) contains supplementary material, which is available to authorized users.

## Background

Globally, approximately 6 million children under 5 years die each year [1]. Further, 300,000 maternal deaths occur every year [2]. Identifying and prioritizing interventions to reduce the burden of morbidity and mortality in women and children is a critical task for governments and ministries of health. It is also a complicated task, requiring decision makers to take into account factors like intervention availability, effectiveness, and cost, the proportion of deaths due to different causes, intervention coverage levels and feasible targets, population changes, and donor preferences.

Global Affairs Canada is funding the development and implementation of the National Evaluation Platform (NEP) in Malawi, Mali, Mozambique, and Tanzania. The NEP is a new rigorous *approach* to compiling and analyzing health and nutrition data from diverse sources. It works with Ministries of Health (MoH), National Institutes of Statistics and other partners to build public sector capacity to address key evaluation questions in maternal, newborn and child health and nutrition (MNCH&N) in their countries, with the goal of improving health programs and planning [3]. In Mali, the NEP is led by the Centre for Research, Study and Documentation for Child Survival, the National Institute for Public Health Research, the National Institute of Statistics, the National Directorate of Health in the Ministry of Health and Public Hygiene, and the Unit for Planning and Statistics/Health Sector, Social Development and Family Promotion. NEP-Mali is advised by a Steering Committee chaired by the Minister of Health and Public Hygiene (MoHPH) and that also includes non-governmental, academic, civil society, and government representatives. A technical working group (TWG) of technical staff from the stakeholder institutions carries out analyses to answer priority evaluation questions identified by the Steering Committee.

To improve health and social outcomes and reduce mortality, especially in women and in children under 5 years, every ten years the government of Mali develops the *Plan Décennal de Développement Sanitaire et Social (PDDSS),* which includes two five year plans, the *Programme de Développement Sanitaire et Social (PRODSS)*. In the first year of NEP implementation in Mali (2014), the country finalized the PDDSS 2014-23 as well as the PRODESS 2014-18 plans [4, 5]. Both documents included targets for maternal, neonatal, and under-5 mortality and stunting and wasting in children under 5 years, as well as a number of intervention coverage targets. As its first task, the NEP-Mali Steering Committee asked the TWG to assess whether the intervention coverage targets in these plans would allow Mali to achieve its MNCH mortality targets, and to assess the effectiveness of alternative intervention packages and targets in achieving these mortality targets. The TWG used the Lives Saved Tool (LiST), a modelling tool that brings together the best available evidence on mortality and intervention effectiveness [6], to conduct this analysis. We present the approach and results here.

## Methods

This study used the Lives Saved Tool (LiST) to conduct a prospective analysis of the effects of various intervention packages on maternal, neonatal, and under-5 mortality in Mali, from 2014 to 2023.

### Setting

**Fig. 1:**
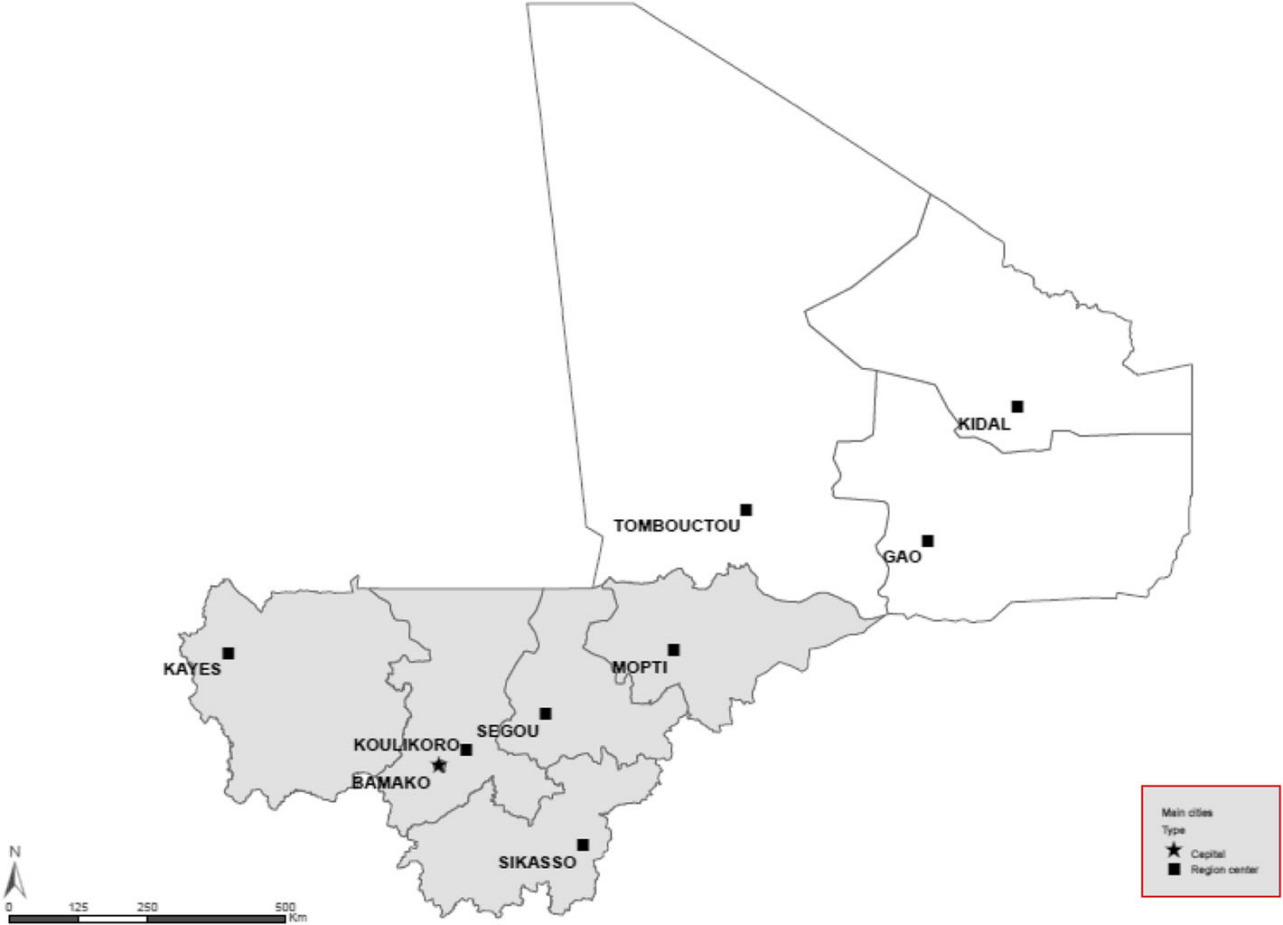
Map of Mali showing (in gray) the five regions and the District of Bamako included in the analysis

Although the PDDSS sets national mortality targets for mothers and children in Mali, our analysis excluded three regions: Gao, Toumbouctou, and Kidal. Due to security issues in the north of Mali, these regions were not included in the 2012 DHS [7], which was the most recent source of coverage data, and therefore had no recent coverage data. The five regions included in our study are Kayes, Koulikoro, Sikasso, Ségou, Mopti and the district of Bamako (Fig. 1).

### Analysis

We conducted this analysis using LiST version 5.07. LiST is a mathematical model that brings together the best available data on population, cause of death, intervention effectiveness, and coverage to estimate the impact of change in intervention coverage on maternal, neonatal, and child mortality [8].

We constructed LiST models, or projections, for both Mali as a whole and for each individual region; in this paper we present only the national results. The national model covered only the five regions included in the 2012 DHS (mentioned above). The starting year for all projections was 2014 and the end year was 2023. All analyses used the number of deaths and the mortality rate at baseline (2014) to calculate the number of deaths averted and the reduction in mortality. We included contraceptive use as an input, but did not calculate deaths averted due to reduced births (decreased population growth arising from reduced fertility rates), as our primary measures of interest were mortality rates, not overall deaths averted.

### Data sources

Baseline coverage estimates (2014) were primarily drawn from the 2012 Mali Demographic and Health Survey (EDSM-2012) [7]. We also used WHO/UNICEF immunization coverage data [9]. We used Stata 13 to recalculate the baseline age-specific and category-specific stunting/wasting indicators required by LiST using the raw Mali 2012 DHS data. Table 1 lists all baseline coverage inputs; for any indicators not included in Table 1 we maintained the default LiST values for Mali in 2014. (Additional file 1 shows all coverage values and nutritional statuses used in the LiST analyses) We used under-five mortality estimates from the United Nations Interagency Group for Child Mortality Estimate (IGME) for 2013 to estimate baseline mortality (Table 2) [10]. We used default LiST values for all other inputs, including cause of death, intervention effectiveness, and population.

**Table 1:** Baseline and endline coverage levels for each of the three projections at national level

Interventions	2014 Baseline (%)	2023 Endline		
		Projection 1 target (%)	Projection 2 target (%)	PRODESS/PDDSS target (%)
Contraceptive prevalence	11	20	20	20
Antenatal care	41	55	50	65
Tetanus toxoid vaccination	42	80	80	
Pregnant women protected by insecticide treated bednets *(ITN)*	73	90	90	
Iron Supplementation	22	30	30	
Malaria case management	0	5	5	
Skilled birth attendance	60	90	90	90
Health facility delivery	60	90	90	
Basic Emergency Obstetric and Newborn Care (BEmOC)	9	30	35	9
Exclusive breastfeeding (0 – 1 month)	59	90	90	
Exclusive breastfeeding (1 – 5 month)	31	70	70	
Clean postnatal practices	16	30	30	
Vitamin A supplementation	61	70	80	
Zinc supplementation	0	4	4	
Improved water source	66	80	80	
Water connection in the home	9	10	10	
Utilization of latrines or toilets	24	30	30	30
Hand washing with soap	17	50	50	
Ownership of ITNs	84	95	95	
DPT-three doses	74	98	98	98
*Hemophilus. influenzae B* (HiB) – three doses	74	98	98	98
*Hepatitis B vaccine* (HepB) – three doses	74	98	98	98
Measles – single dose	72	98	98	98
BCG – single dose	87	98	98	
Rotavirus two doses	0	20	20	
Pneumococcal – three doses	74	98	98	
Polio – three doses	81	98	98	
Thermal care	16	45	45	
Oral antibiotic for newborn	7	20	15	
Vitamin A for Measles treatment	61	70	70	
Newborn sepsis case management	27	55	55	
ORS – oral rehydration solution	37	50	60	
Antibiotic for treatment of dysentery	15	30	50	
Zinc – for treatment of diarrhea	2	30	20	
Oral antibiotic for pneumonia	27	40	50	
Artemisinin for malaria	15	40	50	
Stunting	38	8	15	15
Wasting	13	4	5	5

**Table 2:** National maternal, neonatal, and under-five mortality rates for Mali in 2012, and PDDSS targets for 2023

Mortality rates	Baseline for 2013	PDDSS target in 2023
Newborn	39.4 deaths per 1000 live births [10]	25 deaths per 1000 live births
Under-five	122.7 deaths per 1000 live births [10]	69 deaths per 1000 live births
Maternal	368 deaths per 100,000 live births [7]	146 deaths per 100,000 live births

### Projections

We constructed three LiST projections: one based on the actual PRODESS and PDDSS coverage targets (PRODESS/PDDSS projection) and two with alternative intervention packages and targets (Table 1). The two alternative projections were developed by the Mali NEP TWG during a 5 day workshop in Bamako, Mali in December 2014 and were presented to and approved by the Mali NEP Steering Committee. The interventions included in these projections and their targets were based on the TWG’s knowledge of the Malian health system, intervention effectiveness, and the relevance and feasibility of scaling up different interventions in the Malian context. The first alternative projection (Projection 1) was focused on reducing malnutrition, which is a priority for the Mali MoHPH, and the second (Projection 2) on curative interventions, which are highly effective against the major causes of under-5 mortality in Mali. For the details of the three projections, refer to the Additional file 1.

### PRODESS/PDDSS projection

This projection modeled all coverage interventions with quantitative coverage targets specified by the PRODESS and PDDSS plans. For interventions with targets in both PRODESS and PDDSS, we conducted a linear interpolation of coverage levels from 2014 to the PRODESS target in 2018 and then again from 2018 to the PDDSS target in 2023. For interventions with a 2023 target only, we used linear interpolation from 2014 to 2023. For indicators that only had a target in PRODESS but not in PDDSS, we used linear interpolation from 2014 to 2018 and then duplicated values from 2018 to 2023. For interventions not included in either the PRODESS or PDDSS, we assumed that the coverage of the intervention would not change between 2014 and 2023.

#### Projection 1

This projection set targets for each of the LiST interventions based on the baseline data and the working group’s best estimation of feasible coverage targets, and modeled the effect of reduced stunting and wasting on under-5 mortality (Table 1). The main feature of this projection was large reductions in stunting (from 38.3% to 8%) and wasting (from 12.7% to 4%); we also proposed large increases in the coverage of exclusive breastfeeding.

#### Projection 2

This projection resembled Projection 1 for most interventions but proposed more ambitious targets for curative interventions for young children, including oral rehydration solution, antimalarials, and antibiotics for pneumonia and dysentery, and less ambitious targets for malnutrition (Table 1).

## Results

### Effect on under-5 mortality

**Fig. 2:**
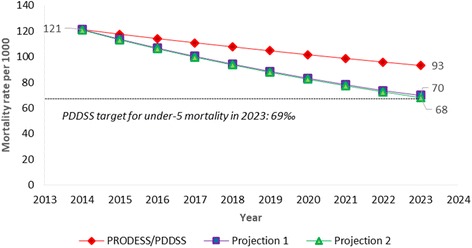
Modeled under-5 mortality rate from 2014 to 2023 in Mali, based on three different LiST projections

Figure 2 shows the estimated changes in the under-five mortality rate from 2014 to 2023 based on the PRODESS/PDDSS projection and projections 1 and 2. The PRODESS/PDDSS projection would reduce mortality from 121 to 93 deaths per thousand live births from 2014 to 2023, saving approximately 125,000 lives over this period (Table 3). The projected mortality level in 2023 would be well above the PDDSS’ under-5 mortality target of 69 deaths per thousand live births. Projections 1 and 2 would both reduce under-5 mortality to a level consistent with the PDDSS target by 2023, saving approximately 240,000 lives over 10 years (Table 3).

**Table 3:** Modeled number of lives saved from 2014 to 2023 in Mali, based on LiST projections

Demographic group	Number of lives saved		
	PRODESS/PDDSS	Projection 1	Projection 2
< 1 month	29,974	63,967	64,641
1-59 months	95,280	177,574	180,490
Mother	3725	3727	3949
Total	128,979	245,268	249,080

### Lives saved by intervention

**Fig. 3:**
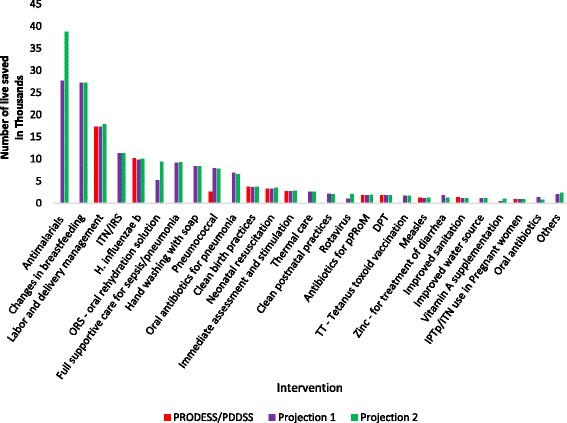
Number of lives saved from 2014 to 2023 in Mali by intervention, based on three different LiST projections

Figure 3 shows the distribution of under-5 lives saved by intervention under each of the three scenarios (not including the deaths prevented through the reduction in stunting and wasting). Of the five interventions responsible for the largest number of lives saved, only two (labor and delivery management and Hib vaccine) were included in the PRODESS and PDDSS. The top two interventions in projections 1 and 2 - antimalarials and breastfeeding practices – together were responsible for 23% and 27% of all under-5 lives saved, respectively, in each projection. The modeled reductions in stunting and wasting accounted for 33%, 27%, and 62%, respectively, of under-5 lives saved in Projection 1, Projection 2, and the PRODESS/PDDSS projection (data not shown). The number of lives saved by intervention and by target are available in the Additional file 2.

### Effect on neonatal mortality

**Fig. 4:**
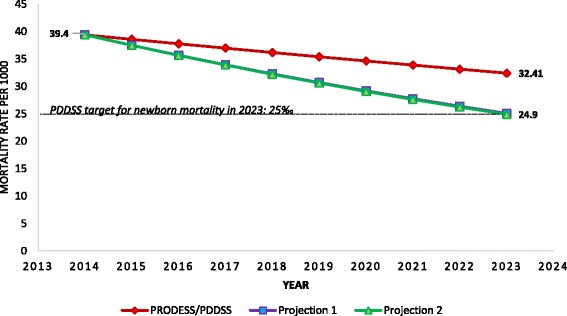
Modeled neonatal mortality rate from 2014 to 2023 in Mali, based on three different LiST projections

Figure 4 shows the modeled changes in neonatal mortality from 2014 to 2023 according to our three LiST projections. The PRODESS/PDDSS projection would not allow Mali to reach its mortality target for newborns (25 deaths per 1000 live births). Projections 1 and 2 both resulted in modeled levels of neonatal mortality of 25 per 1000 live births in 2023, meeting the PDDSS mortality target.

### Effect on maternal mortality

**Fig. 5:**
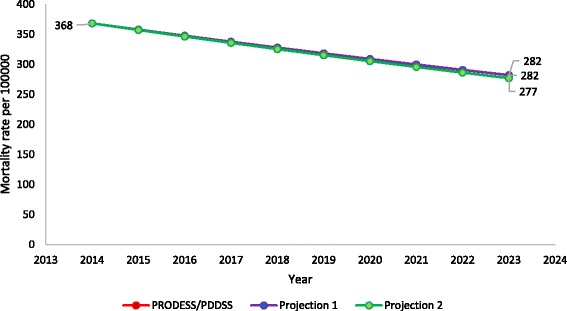
Modeled maternal mortality ratio from 2014 to 2023 in Mali, based on three different LiST projections

The modeled maternal mortality ratios from our three LiST projections are shown in Fig. 5. The three projections resulted in mortality reductions of between 86 and 91 deaths per 100,000 live births over 10 years. The endline mortality ratios between 277 and 282 per 100,000 live births were far above the PDDSS mortality target of 146.

## Discussion

This analysis had several major findings. First, we found that achieving the coverage targets for the MNCH&N interventions in the 2014-23 PDDSS and 2014-18 PRODESS would likely not allow Mali to achieve its mortality targets. In fact, the under-5 mortality rate for 2023 modeled using the PDDSS and PRODESS coverage rates was 30% above the targeted rate. A related finding is that many of the most effective child health interventions (insecticide treated bednets, breastfeeding practices, etc.) had not been included among the PRODESS or PDDSS targets.

Second, the NEP-Mali team was able to identify two packages of MNCH&N interventions (and targets) that achieved under-5 and neonatal mortality rates at, or very near, the PDDSS targets. They also saved more newborn, maternal and under-5 lives in than PRODESS/PDDSS (Webannex 4, 5 and 6). While one of these packages required very large reductions in malnutrition, the other paired modest increases in the coverage of curative and childbirth interventions with more moderate reductions in malnutrition.

Finally, none of the projections produced by NEP-Mali led to reductions in maternal mortality large enough to achieve the PDDSS target; in fact, all three projections had approximately the same estimated effect on maternal mortality. This result highlights the need for more attention to the interventions for mothers and for the identification of potential strategies to improve those interventions.

Our analysis had a number of limitations, many of which were related to the projection inputs. While we used the most recent high-quality coverage data available for Mali – the 2012-13 DHS [7] – these data represent a period slightly prior to our desired baseline of 2014. In addition, the DHS did not include the three northernmost regions in Mali, so our projections were limited to the central and southern regions of the country. If the northern areas had poorer coverage levels than the rest of the country in 2012, then their exclusion might have reduced the modeled reduction in mortality; in other words, we would have under-estimated the effects of all three intervention packages on mortality. However, the population in the excluded areas is relatively small compared to the rest of the country. In fact those areas represent 8.8% of the total population of Mali [11], so the impact of their exclusion from the model is likely minimal.

Another data-related limitation is the fact that not all LiST interventions are measured in the DHS. Thus, for some LiST interventions, we did not have a good baseline estimate and had to use proxies. For nutrition, where many of the interventions of interest (counseling on breastfeeding and complementary feeding, treatment of acute malnutrition) are not measured in DHS, we opted to directly enter the baseline rates of malnutrition and the targeted endline rates, instead of modelling the effect of the nutrition interventions.

Finally, the results of our LiST projections are based on the assumption that the modeled coverage targets will be achieved. This analysis did not address the inputs and processes needed to achieve the coverage targets, although the NEP working group, which included epidemiologists, nutritionists, and public health doctors, tried to select targets that it deemed achievable. Further work by the MoHPH would be needed to determine how to reach these targets.

## Conclusions

The results of this analysis were presented to the NEP Steering Committee and disseminated in 2015 [12]. The MoHPH indicated that they planned to use the findings to revise the coverage and mortality targets in the PDDSS and PRODESS, with the goal of having mortality targets consistent with the intervention coverage targets in the report. The MoHPH has planned to conduct LiST training for its regional staff, with technical assistance from NEP-Mali and LiST, to allow them to use LiST for evidence-based regional health planning. This analysis provides an example of how LiST can be used to facilitate evidence-based planning in countries, and could also be done in countries without an NEP.

## Additional files

Additional file 1: interventions and coverage of three LiST analyses, Word Document. (DOCX 43 kb)

Additional file 2: Results from LiST, Word Document. (DOCX 17 kb)
