# Supplementary material for: Using the Lives Saved Tool to aid country planning in meeting mortality targets: a case study from Mali
Source: BMC Public Health. 2017 Nov 7;17(Suppl 4):777. doi: 10.1186/s12889-017-4749-y (PMC5688424; doi:10.1186/s12889-017-4749-y)
Supplement: Supplementary file 1 — interventions and coverage of three LiST analyses, Word Document. (DOCX 43 kb) [file 12889_2017_4749_MOESM1_ESM.docx]

# Additional file 1- interventions and coverage of three LiST analyses

**Table 1** Projection 1 interventions and coverage from 2014 to 2023

| **Interventions** | **Years of decennial plan implementation** | | | | | | | | | |
| --- | --- | --- | --- | --- | --- | --- | --- | --- | --- | --- |
|  | *2014*  *baseline** | *2015* | *2016* | *2017* | *2018* | *2019* | *2020* | *2021* | *2022* | *2023*  *Endline* |
| **Periconceptual** |  |  |  |  |  |  |  |  |  |  |
| Prevalence contraceptive | 11 | 12 | 13 | 14 | 15 | 16 | 17 | 18 | 19 | 20 |
| **Pregnancy** |  |  |  |  |  |  |  |  |  |  |
| Antenatal care | 41 | 43 | 44 | 46 | 47 | 49 | 50 | 52 | 53 | 55 |
| Tetanus toxoid vaccination | 42.3 | 46.5 | 50.7 | 54.9 | 59.1 | 63.2 | 67.4 | 71.6 | 75.8 | 80 |
| Pregnant women protected by ITN or IPTp | 73.2 | 75.1 | 76.9 | 78.8 | 80.7 | 82.5 | 84.4 | 86.3 | 88.1 | 90 |
| Iron Supplementation | 22 | 22.9 | 23.8 | 24.7 | 25.6 | 26.5 | 27.3 | 28.2 | 29.1 | 30 |
| Malaria case management | 0 | 0.6 | 1.1 | 1.7 | 2.2 | 2.8 | 3.3 | 3.9 | 4.4 | 5 |
| **Childbirth** |  |  |  |  |  |  |  |  |  |  |
| Skilled birth attendance | 60 | 63 | 67 | 70 | 73 | 77 | 80 | 83 | 87 | 90 |
| Health facility delivery | 60 | 63.3 | 66.7 | 70 | 73.3 | 76.7 | 80 | 83.3 | 86.7 | 90 |
| BEmoC | 9 | 11.3 | 13.7 | 16 | 18.3 | 20.7 | 23 | 25.3 | 27.7 | 30 |
| **Breastfeeding** |  |  |  |  |  |  |  |  |  |  |
| **0 - 1 month** |  |  |  |  |  |  |  |  |  |  |
| Exclusive breastfeeding | 59 | 63.0 | 66.0 | 70.0 | 73.0 | 76.0 | 80.0 | 83.0 | 87.0 | 90 |
| Predominant breastfeeding | 22 | 20.7 | 19.1 | 17.4 | 15.8 | 14.1 | 12.5 | 10.8 | 9.2 | 8 |
| Partial breastfeeding | 14 | 12.7 | 11.3 | 9.9 | 8.5 | 7.1 | 5.7 | 4.3 | 2.9 | 2 |
| Not breastfeeding | 4 | 3.9 | 3.5 | 3.2 | 2.8 | 2.5 | 2.1 | 1.7 | 1.4 | 1 |
| **1 - 5 months** |  |  |  |  |  |  |  |  |  |  |
| Exclusive breastfeeding | 31 | 36.0 | 40.0 | 44.0 | 49.0 | 53.0 | 57.0 | 61.0 | 66.0 | 70 |
| Predominant breastfeeding | 38 | 35.5 | 33.1 | 30.8 | 28.4 | 26.0 | 23.6 | 21.3 | 18.9 | 17 |
| Partial breastfeeding | 27 | 24.8 | 23.2 | 21.5 | 19.9 | 18.2 | 16.6 | 14.9 | 13.3 | 12 |
| Not breastfeeding | 4 | 4.0 | 3.8 | 3.5 | 3.2 | 3.0 | 2.7 | 2.4 | 2.2 | 2 |
| **6 - 11 months** |  |  |  |  |  |  |  |  |  |  |
| Any breastfeeding | 96 | 96 | 97 | 97 | 97 | 97 | 97 | 98 | 98 | 98 |
| Not breastfeeding | 4 | 4 | 4 | 3 | 3 | 3 | 3 | 2 | 2 | 2 |
| **12 - 23 months** |  |  |  |  |  |  |  |  |  |  |
| Any breastfeeding | 81 | 81 | 82 | 82 | 83 | 83 | 84 | 84 | 85 | 85 |
| Not breastfeeding | 19 | 19 | 18 | 18 | 17 | 17 | 16 | 16 | 16 | 15 |
| **Preventive** |  |  |  |  |  |  |  |  |  |  |
| **Postnatal care** |  |  |  |  |  |  |  |  |  |  |
| Clean postnatal practices | 16 | 17.2 | 18.8 | 20.4 | 22 | 23.6 | 25.2 | 26.8 | 28.4 | 30 |
| Complementary feeding |  |  |  |  |  |  |  |  |  |  |
| Vitamin A supplementation | 60.8 | 61.9 | 62.9 | 63.9 | 64.9 | 65.9 | 66.9 | 68 | 69 | 70 |
| Zinc supplementation | 0 | 0.4 | 0.9 | 1.3 | 1.8 | 2.2 | 2.7 | 3.1 | 3.6 | 4 |
| **WASH** |  |  |  |  |  |  |  |  |  |  |
| Improved water source | 66.4 | 67.9 | 69.4 | 70.9 | 72.4 | 73.9 | 75.5 | 77 | 78.5 | 80 |
| Water connection in the home | 8.8 | 8.9 | 9 | 9.2 | 9.3 | 9.4 | 9.6 | 9.7 | 9.9 | 10 |
| Utilization of latrines or toilets | 23.8 | 24.5 | 25.2 | 25.9 | 26.6 | 27.2 | 27.9 | 28.6 | 29.3 | 30 |
| Hand washing with soap | 17 | 20.7 | 24.3 | 28 | 31.7 | 35.3 | 39 | 42.7 | 46.3 | 50 |
| Ownership of ITN | 84.4 | 85.6 | 86.7 | 87.9 | 89.1 | 90.3 | 91.5 | 92.6 | 93.8 | 95 |
| **Vaccines** |  |  |  |  |  |  |  |  |  |  |
| DPT-three doses | 74 | 76.7 | 79.3 | 82 | 84.7 | 87.3 | 90 | 92.7 | 95.3 | 98 |
| H. influenza – three doses | 74 | 76.7 | 79.3 | 82 | 84.7 | 87.3 | 90 | 92.7 | 95.3 | 98 |
| HepB – three doses | 74 | 76.7 | 79.3 | 82 | 84.7 | 87.3 | 90 | 92.7 | 95.3 | 98 |
| Measles – single dose | 72 | 75 | 78 | 81 | 84 | 86 | 89 | 92 | 95 | 98 |
| BCG – single dose | 87 | 88 | 89 | 91 | 92 | 93 | 94 | 96 | 97 | 98 |
| Pneumococcal – three doses | 74 | 76.7 | 79.3 | 82 | 84.7 | 87.3 | 90 | 92.7 | 95.3 | 98 |
| Polio – three doses | 81 | 82.9 | 84.8 | 86.7 | 88.6 | 90.4 | 92.3 | 94.2 | 96.1 | 98 |
| Rotavirus | 0 | 2 | 4 | 7 | 9 | 11 | 13 | 16 | 18 | 20 |
| **Curative** |  |  |  |  |  |  |  |  |  |  |
| Thermal care | 15.6 | 18.9 | 22.1 | 25.4 | 28.7 | 31.9 | 35.2 | 38.5 | 41.7 | 45 |
| Oral antibiotic for newborn | 7 | 8.4 | 9.8 | 11.3 | 12.8 | 14.2 | 15.7 | 17.1 | 18.6 | 20 |
| Vitamin A for Measles treatment | 60.8 | 61.9 | 62.9 | 63.9 | 64.9 | 65.9 | 66.9 | 68 | 69 | 70 |
| Newborn sepsis case management | 27.5 | 30.6 | 33.6 | 36.7 | 39.7 | 42.8 | 45.8 | 48.9 | 51.9 | 55 |
| **Diarrhea** |  |  |  |  |  |  |  |  |  |  |
| ORS – oral rehydration solution | 36.8 | 38.3 | 39.7 | 41.2 | 42.7 | 44.1 | 45.6 | 47.1 | 48.5 | 50 |
| Antibiotic for treatment of dysentery | 15.4 | 17 | 18.6 | 20.2 | 21.9 | 23.5 | 25.1 | 26.7 | 28.4 | 30 |
| Zinc – for treatment of diarrhea | 2.1 | 5.2 | 8.3 | 11.4 | 14.5 | 17.6 | 20.7 | 23.8 | 26.9 | 30 |
| Oral antibiotic for pneumonia | 26.7 | 29.8 | 33 | 36.1 | 39.3 | 42.4 | 45.6 | 48.7 | 51.9 | 40 |
| Artemisinin for malaria | 15 | 17.8 | 20.6 | 23.4 | 26.1 | 28.9 | 31.7 | 34.5 | 37.2 | 40 |
| **Stunting*** | 37.3 | 34.0 | 30.8 | 27.5 | 24.3 | 21.0 | 17.8 | 14.5 | 11.3 | 8 |
| <1 month |  |  |  |  |  |  |  |  |  |  |
| (less than -1 sd | 68.7 | 68.4 | 68.0 | 67.7 | 67.4 | 67.0 | 66.7 | 66.4 | 66.0 | 65.7 |
| (between -1 et -2 sd) | 16.1 | 17.2 | 18.4 | 19.5 | 20.6 | 21.8 | 22.9 | 24.0 | 25.2 | 26.3 |
| (between -2 et -3 sd) | 10.2 | 9.9 | 9.5 | 9.2 | 8.9 | 8.5 | 8.2 | 7.9 | 7.5 | 7.2 |
| (less than -3 sd) | 5.0 | 4.5 | 4.1 | 3.6 | 3.1 | 2.7 | 2.2 | 1.7 | 1.3 | 0.8 |
| 1-5 months |  |  |  |  |  |  |  |  |  |  |
| (less than -1 sd | 68.7 | 68.4 | 68.0 | 67.7 | 67.4 | 67.0 | 66.7 | 66.4 | 66.0 | 65.7 |
| (between -1 et -2 sd) | 16.1 | 17.2 | 18.4 | 19.5 | 20.6 | 21.8 | 22.9 | 24.0 | 25.2 | 26.3 |
| (between -2 et -3 sd) | 10.2 | 9.9 | 9.5 | 9.2 | 8.9 | 8.5 | 8.2 | 7.9 | 7.5 | 7.2 |
| (less than -3 sd) | 5.0 | 4.5 | 4.1 | 3.6 | 3.1 | 2.7 | 2.2 | 1.7 | 1.3 | 0.8 |
| 6-11 months |  |  |  |  |  |  |  |  |  |  |
| (less than -1 sd | 62.9 | 63.2 | 63.5 | 63.8 | 64.1 | 64.5 | 64.8 | 65.1 | 65.4 | 65.7 |
| (between -1 et -2 sd) | 20.3 | 20.9 | 21.6 | 22.3 | 23.0 | 23.6 | 24.3 | 25.0 | 25.6 | 26.3 |
| (between -2 et -3 sd) | 9.0 | 8.8 | 8.6 | 8.4 | 8.2 | 8.0 | 7.8 | 7.6 | 7.4 | 7.2 |
| (less than -3 sd) | 7.8 | 7.0 | 6.3 | 5.5 | 4.7 | 3.9 | 3.1 | 2.4 | 1.6 | 0.8 |
| 12-23 months |  |  |  |  |  |  |  |  |  |  |
| (less than -1 sd | 37.1 | 40.2 | 43.4 | 46.6 | 49.8 | 53.0 | 56.2 | 59.3 | 62.5 | 65.7 |
| (between -1 et -2 sd) | 25.1 | 25.2 | 25.3 | 25.5 | 25.6 | 25.7 | 25.9 | 26.0 | 26.2 | 26.3 |
| (between -2 et -3 sd) | 19.3 | 17.9 | 16.6 | 15.2 | 13.9 | 12.6 | 11.2 | 9.9 | 8.5 | 7.2 |
| (less than -3 sd) | 18.7 | 16.7 | 14.7 | 12.7 | 10.7 | 8.7 | 6.8 | 4.8 | 2.8 | 0.8 |
| 24-59 months |  |  |  |  |  |  |  |  |  |  |
| (less than -1 sd | 32.1 | 35.8 | 39.5 | 43.3 | 47.0 | 50.7 | 54.5 | 58.2 | 62.0 | 65.7 |
| (between -1 et -2 sd) | 22.9 | 23.2 | 23.6 | 24.0 | 24.4 | 24.8 | 25.2 | 25.5 | 25.9 | 26.3 |
| (between -2 et -3 sd) | 22.1 | 20.4 | 18.8 | 17.1 | 15.5 | 13.8 | 12.2 | 10.5 | 8.9 | 7.2 |
| (less than -3 sd) | 23.0 | 20.5 | 18.1 | 15.6 | 13.1 | 10.7 | 8.2 | 5.7 | 3.3 | 0.8 |
| **Wasting*** | 13.0 | 12.0 | 11.0 | 10.0 | 9.0 | 8.0 | 7.0 | 6.0 | 5.0 | 4 |
| <1 month |  |  |  |  |  |  |  |  |  |  |
| (less than -1 sd | 60.5 | 62.4 | 64.2 | 66.1 | 68.0 | 69.9 | 71.8 | 73.6 | 75.5 | 77.4 |
| (between -1 et -2 sd) | 21.5 | 21.2 | 20.8 | 20.5 | 20.2 | 19.9 | 19.6 | 19.2 | 18.9 | 18.6 |
| (between -2 et -3 sd) | 7.4 | 7.0 | 6.6 | 6.2 | 5.8 | 5.4 | 4.9 | 4.5 | 4.1 | 3.7 |
| (less than -3 sd) | 10.6 | 9.5 | 8.3 | 7.2 | 6.0 | 4.9 | 3.7 | 2.6 | 1.4 | 0.3 |
| 1-5 months |  |  |  |  |  |  |  |  |  |  |
| (less than -1 sd | 60.5 | 62.4 | 64.2 | 66.1 | 68.0 | 69.9 | 71.8 | 73.6 | 75.5 | 77.4 |
| (between -1 et -2 sd) | 21.5 | 21.2 | 20.8 | 20.5 | 20.2 | 19.9 | 19.6 | 19.2 | 18.9 | 18.6 |
| (between -2 et -3 sd) | 7.4 | 7.0 | 6.6 | 6.2 | 5.8 | 5.4 | 4.9 | 4.5 | 4.1 | 3.7 |
| (less than -3 sd) | 10.6 | 9.5 | 8.3 | 7.2 | 6.0 | 4.9 | 3.7 | 2.6 | 1.4 | 0.3 |
| 6-11 months |  |  |  |  |  |  |  |  |  |  |
| (less than -1 sd | 55.6 | 58.0 | 60.4 | 62.9 | 65.3 | 67.7 | 70.1 | 72.6 | 75.0 | 77.4 |
| (between -1 et -2 sd) | 24.9 | 24.2 | 23.5 | 22.8 | 22.1 | 21.4 | 20.7 | 20.0 | 19.3 | 18.6 |
| (between -2 et -3 sd) | 13.5 | 12.4 | 11.3 | 10.2 | 9.2 | 8.1 | 7.0 | 5.9 | 4.8 | 3.7 |
| (less than -3 sd) | 6.0 | 5.3 | 4.7 | 4.1 | 3.5 | 2.8 | 2.2 | 1.6 | 0.9 | 0.3 |
| 12-23 months |  |  |  |  |  |  |  |  |  |  |
| (less than -1 sd | 58.4 | 60.5 | 62.6 | 64.8 | 66.9 | 69.0 | 71.1 | 73.2 | 75.3 | 77.4 |
| (between -1 et -2 sd) | 23.1 | 22.6 | 22.1 | 21.6 | 21.1 | 20.6 | 20.1 | 19.6 | 19.1 | 18.6 |
| (between -2 et -3 sd) | 11.7 | 10.8 | 9.9 | 9.0 | 8.1 | 7.3 | 6.4 | 5.5 | 4.6 | 3.7 |
| (less than -3 sd) | 6.7 | 6.0 | 5.3 | 4.6 | 3.9 | 3.2 | 2.4 | 1.7 | 1.0 | 0.3 |
| 24-59 months |  |  |  |  |  |  |  |  |  |  |
| (less than -1 sd | 73.3 | 73.4 | 73.9 | 74.4 | 74.9 | 75.4 | 75.9 | 76.4 | 76.9 | 77.4 |
| (between -1 et -2 sd) | 17.5 | 17.9 | 18.0 | 18.0 | 18.1 | 18.2 | 18.3 | 18.4 | 18.5 | 18.6 |
| (between -2 et -3 sd) | 5.5 | 5.4 | 5.2 | 5.0 | 4.8 | 4.5 | 4.3 | 4.1 | 3.9 | 3.7 |
| (less than -3 sd) | 3.7 | 3.4 | 3.0 | 2.6 | 2.2 | 1.8 | 1.5 | 1.1 | 0.7 | 0.3 |

* 2014 baseline values are estimated based on 2012-13 DHS data, assuming no change from 2012-13 to 2014

**Table 2** Projection 2 interventions and coverage from 2014 to 2023

| **Intervention** | **Years of decennial plan implementation** | | | | | | | | | |
| --- | --- | --- | --- | --- | --- | --- | --- | --- | --- | --- |
|  | *2014*  *baseline** | *2015* | *2016* | *2017* | *2018* | *2019* | *2020* | *2021* | *2022* | *2023*  *Endline* |
| **Periconceptual** |  |  |  |  |  |  |  |  |  |  |
| Prevalence contraceptive | 11 | 12 | 13 | 14 | 15 | 16 | 17 | 18 | 19 | 20 |
| **Pregnancy** |  |  |  |  |  |  |  |  |  |  |
| Antenatal care | 41.3 | 42.2 | 43.2 | 44.2 | 45.1 | 46.1 | 47.1 | 48.1 | 49 | 50 |
| Tetanus toxoid vaccination | 42.3 | 46.5 | 50.7 | 54.9 | 59.1 | 63.2 | 67.4 | 71.6 | 75.8 | 80 |
| Pregnant women protected by ITN or IPTp | 73.2 | 75.1 | 76.9 | 78.8 | 80.7 | 82.5 | 84.4 | 86.3 | 88.1 | 90 |
| Iron supplementation | 22 | 22.9 | 23.8 | 24.7 | 25.6 | 26.5 | 27.3 | 28.2 | 29.1 | 30 |
| Malaria case management | 0 | 0.6 | 1.1 | 1.7 | 2.2 | 2.8 | 3.3 | 3.9 | 4.4 | 5 |
| **Childbirth** |  |  |  |  |  |  |  |  |  |  |
| Skilled birth attendance | 60 | 63.3 | 66.7 | 70 | 73.3 | 76.7 | 80 | 83.3 | 86.7 | 90 |
| Health facility delivery | 60 | 63.3 | 66.7 | 70 | 73.3 | 76.7 | 80 | 83.3 | 86.7 | 90 |
| BEmoC | 9 | 11.9 | 14.8 | 17.7 | 20.6 | 23.4 | 26.3 | 29.2 | 32.1 | 35 |
| **Breastfeeding** |  |  |  |  |  |  |  |  |  |  |
| **0 - 1 month** |  |  |  |  |  |  |  |  |  |  |
| Exclusive breastfeeding | 59 | 63.0 | 66.0 | 70.0 | 73.0 | 76.0 | 80.0 | 83.0 | 87.0 | 90 |
| Predominant breastfeeding | 22 | 20.7 | 19.1 | 17.4 | 15.8 | 14.1 | 12.5 | 10.8 | 9.2 | 8 |
| Partial breastfeeding | 14 | 12.7 | 11.3 | 9.9 | 8.5 | 7.1 | 5.7 | 4.3 | 2.9 | 2 |
| Not breastfeeding | 4 | 3.9 | 3.5 | 3.2 | 2.8 | 2.5 | 2.1 | 1.7 | 1.4 | 1 |
| **1 - 5 months** |  |  |  |  |  |  |  |  |  |  |
| Exclusive breastfeeding | 31 | 36 | 40 | 44 | 49 | 53 | 57 | 61 | 66 | 70 |
| Predominant breastfeeding | 38 | 36 | 33 | 31 | 28 | 26 | 24 | 21 | 19 | 17 |
| Partial breastfeeding | 27 | 25 | 23 | 22 | 20 | 18 | 17 | 15 | 13 | 12 |
| Not breastfeeding | 4 | 4 | 4 | 4 | 3 | 3 | 3 | 2 | 2 | 2 |
| **6 - 11 months** |  |  |  |  |  |  |  |  |  |  |
| Any breastfeeding | 96 | 96 | 97 | 97 | 97 | 97 | 97 | 98 | 98 | 98 |
| Not breastfeeding | 4 | 4 | 4 | 3 | 3 | 3 | 3 | 2 | 2 | 2 |
| **12 - 23 months** |  |  |  |  |  |  |  |  |  |  |
| Any breastfeeding | 81 | 81 | 82 | 82 | 83 | 83 | 84 | 84 | 85 | 85 |
| Not breastfeeding | 19 | 19 | 18 | 18 | 17 | 17 | 16 | 16 | 16 | 15 |
| **Preventive** |  |  |  |  |  |  |  |  |  |  |
| **Postnatal care** |  |  |  |  |  |  |  |  |  |  |
| Clean postnatal practices | 15.6 | 17.2 | 18.8 | 20.4 | 22 | 23.6 | 25.2 | 26.8 | 28.4 | 30 |
| **Complementary feeding** |  |  |  |  |  |  |  |  |  |  |
| Vitamin A supplementation | 60.8 | 63 | 65.1 | 67.2 | 69.4 | 71.5 | 73.6 | 75.7 | 77.9 | 80 |
| Zinc supplementation | 0 | 0.4 | 0.9 | 1.3 | 1.8 | 2.2 | 2.7 | 3.1 | 3.6 | 4 |
| **WASH** |  |  |  |  |  |  |  |  |  |  |
| Improved water source | 66.4 | 67.9 | 69.4 | 70.9 | 72.4 | 73.9 | 75.5 | 77 | 78.5 | 80 |
| Water connection in the home | 8.8 | 8.9 | 9 | 9.2 | 9.3 | 9.4 | 9.6 | 9.7 | 9.9 | 10 |
| Utilization of latrines or toilets | 23.8 | 24.5 | 25.2 | 25.9 | 26.6 | 27.2 | 27.9 | 28.6 | 29.3 | 30 |
| Hand washing with soap | 17 | 20.7 | 24.3 | 28 | 31.7 | 35.3 | 39 | 42.7 | 46.3 | 50 |
| Ownership of ITN | 84.4 | 85.6 | 86.7 | 87.9 | 89.1 | 90.3 | 91.5 | 92.6 | 93.8 | 95 |
| **Vaccines** |  |  |  |  |  |  |  |  |  |  |
| DPT-three doses | 74 | 76.7 | 79.3 | 82 | 84.7 | 87.3 | 90 | 92.7 | 95.3 | 98 |
| H. influenza – three doses | 74 | 76.7 | 79.3 | 82 | 84.7 | 87.3 | 90 | 92.7 | 95.3 | 98 |
| HepB – three doses | 74 | 76.7 | 79.3 | 82 | 84.7 | 87.3 | 90 | 92.7 | 95.3 | 98 |
| Measles – single dose | 72 | 75 | 78 | 81 | 84 | 86 | 89 | 92 | 95 | 98 |
| BCG – single dose | 87 | 88 | 89 | 91 | 92 | 93 | 94 | 96 | 97 | 98 |
| Rotavirus | 0 | 2 | 4 | 7 | 9 | 11 | 13 | 16 | 18 | 20 |
| Pneumococcal – three doses | 74 | 75.8 | 77.6 | 79.3 | 81.1 | 82.9 | 84.7 | 86.4 | 88.2 | 90 |
| Polio – three doses | 81 | 82.9 | 84.8 | 86.7 | 88.6 | 90.4 | 92.3 | 94.2 | 96.1 | 98 |
| **Curative** |  |  |  |  |  |  |  |  |  |  |
| Thermal care | 15.6 | 18.9 | 22.1 | 25.4 | 28.7 | 31.9 | 35.2 | 38.5 | 41.7 | 45 |
| Oral antibiotic for newborn | 7 | 7.8 | 8.7 | 9.6 | 10.5 | 11.4 | 12.3 | 13.2 | 14.1 | 15 |
| Vitamin A for measles treatment | 60.8 | 61.9 | 62.9 | 63.9 | 64.9 | 65.9 | 66.9 | 68 | 69 | 70 |
| Sepsis management in newborn | 27.5 | 30.6 | 33.6 | 36.7 | 39.7 | 42.8 | 45.8 | 48.9 | 51.9 | 55 |
| **Diarrhea** |  |  |  |  |  |  |  |  |  |  |
| ORS – oral rehydration solution | 36.8 | 39.4 | 42 | 44.5 | 47.1 | 49.7 | 52.3 | 54.8 | 57.4 | 60 |
| Antibiotic for treatment of dysentery | 15.4 | 19.2 | 23.1 | 26.9 | 30.8 | 34.6 | 38.5 | 42.3 | 46.2 | 50 |
| Zinc – for treatment of diarrhea | 2.1 | 4.1 | 6.1 | 8.1 | 10.1 | 12.1 | 14 | 16 | 18 | 20 |
| Oral antibiotic for pneumonia | 26.7 | 29.3 | 31.9 | 34.5 | 37 | 39.6 | 42.2 | 44.8 | 47.4 | 50 |
| Artemisinin for malaria | 15 | 18.9 | 22.8 | 26.7 | 30.6 | 34.5 | 38.3 | 42.2 | 46.1 | 50 |
| **Stunting** | 37.3 | 34.8 | 32.3 | 29.8 | 27.4 | 24.9 | 22.4 | 19.9 | 17.5 | 15 |
| <1 month |  |  |  |  |  |  |  |  |  |  |
| (less than -1 sd | 68.7 | 68.7 | 68.7 | 68.7 | 68.7 | 68.7 | 68.7 | 68.7 | 68.7 | 68.7 |
| (between -1 et -2 sd) | 16.1 | 16.1 | 16.1 | 16.2 | 16.2 | 16.2 | 16.2 | 16.3 | 16.3 | 16.3 |
| (between -2 et -3 sd) | 10.2 | 10.5 | 10.8 | 11.1 | 11.4 | 11.7 | 12.0 | 12.3 | 12.6 | 12.9 |
| (less than -3 sd) | 5.0 | 4.7 | 4.3 | 4.0 | 3.7 | 3.4 | 3.0 | 2.7 | 2.4 | 2.1 |
| 1-5 months |  |  |  |  |  |  |  |  |  |  |
| (less than -1 sd | 68.7 | 68.7 | 68.7 | 68.7 | 68.7 | 68.7 | 68.7 | 68.7 | 68.7 | 68.72 |
| (between -1 et -2 sd) | 16.1 | 16.1 | 16.1 | 16.2 | 16.2 | 16.2 | 16.2 | 16.3 | 16.3 | 16.3 |
| (between -2 et -3 sd) | 10.2 | 10.5 | 10.8 | 11.1 | 11.4 | 11.7 | 12.0 | 12.3 | 12.6 | 12.9 |
| (less than -3 sd) | 5.0 | 4.7 | 4.3 | 4.0 | 3.7 | 3.4 | 3.0 | 2.7 | 2.4 | 2.08 |
| 6-11 months |  |  |  |  |  |  |  |  |  |  |
| (less than -1 sd | 62.9 | 62.9 | 62.9 | 62.9 | 62.9 | 63.0 | 63.0 | 63.0 | 63.0 | 63 |
| (between -1 et -2 sd) | 20.3 | 20.5 | 20.7 | 20.9 | 21.0 | 21.2 | 21.4 | 21.6 | 21.8 | 22 |
| (between -2 et -3 sd) | 9.0 | 9.5 | 9.9 | 10.3 | 10.7 | 11.2 | 11.6 | 12.0 | 12.5 | 12.9 |
| (less than -3 sd) | 7.8 | 7.2 | 6.5 | 5.9 | 5.3 | 4.6 | 4.0 | 3.4 | 2.7 | 2.1 |
| 12-23 months |  |  |  |  |  |  |  |  |  |  |
| (less than -1 sd | 37.1 | 38.6 | 40.2 | 41.8 | 43.4 | 45.0 | 46.6 | 48.2 | 49.8 | 51.4 |
| (between -1 et -2 sd) | 25.1 | 26.0 | 27.0 | 27.9 | 28.9 | 29.8 | 30.8 | 31.7 | 32.7 | 33.6 |
| (between -2 et -3 sd) | 19.3 | 18.5 | 17.8 | 17.1 | 16.4 | 15.7 | 15.0 | 14.3 | 13.6 | 12.9 |
| (less than -3 sd) | 18.7 | 16.8 | 15.0 | 13.1 | 11.3 | 9.5 | 7.6 | 5.8 | 3.9 | 2.1 |
| 24-59 months |  |  |  |  |  |  |  |  |  |  |
| (less than -1 sd | 32.1 | 34.2 | 36.4 | 38.5 | 40.7 | 42.8 | 45.0 | 47.1 | 49.3 | 51.4 |
| (between -1 et -2 sd) | 22.9 | 24.1 | 25.2 | 26.4 | 27.6 | 28.8 | 30.0 | 31.2 | 32.4 | 33.6 |
| (between -2 et -3 sd) | 22.1 | 21.1 | 20.1 | 19.0 | 18.0 | 17.0 | 16.0 | 14.9 | 13.9 | 12.9 |
| (less than -3 sd) | 23.0 | 20.7 | 18.3 | 16.0 | 13.7 | 11.4 | 9.1 | 6.7 | 4.4 | 2.1 |
| **Wasting** | 13.0 | 12.1 | 11.2 | 10.3 | 9.4 | 8.5 | 7.7 | 6.8 | 5.9 | 5. |
| <1 month |  |  |  |  |  |  |  |  |  |  |
| (less than -1 sd | 60.5 | 62.0 | 63.5 | 65.0 | 66.5 | 68.0 | 69.5 | 71.0 | 72.5 | 74 |
| (between -1 et -2 sd) | 21.5 | 21.4 | 21.4 | 21.3 | 21.3 | 21.2 | 21.2 | 21.1 | 21.1 | 21 |
| (between -2 et -3 sd) | 7.4 | 7.1 | 6.8 | 6.5 | 6.2 | 5.9 | 5.5 | 5.2 | 4.9 | 4.6 |
| (less than -3 sd) | 10.6 | 9.5 | 8.4 | 7.2 | 6.1 | 4.9 | 3.8 | 2.7 | 1.5 | 0.4 |
| 1-5 months |  |  |  |  |  |  |  |  |  |  |
| (less than -1 sd | 60.5 | 62.0 | 63.5 | 65.0 | 66.5 | 68.0 | 69.5 | 71.0 | 72.5 | 74 |
| (between -1 et -2 sd) | 21.5 | 21.4 | 21.4 | 21.3 | 21.3 | 21.2 | 21.2 | 21.1 | 21.1 | 21 |
| (between -2 et -3 sd) | 7.4 | 7.1 | 6.8 | 6.5 | 6.2 | 5.9 | 5.5 | 5.2 | 4.9 | 4.6 |
| (less than -3 sd) | 10.6 | 9.5 | 8.4 | 7.2 | 6.1 | 4.9 | 3.8 | 2.7 | 1.5 | 0.4 |
| 6-11 months |  |  |  |  |  |  |  |  |  |  |
| (less than -1 sd | 55.6 | 57.6 | 59.7 | 61.7 | 63.8 | 65.8 | 67.9 | 69.9 | 72.0 | 74 |
| (between -1 et -2 sd) | 24.9 | 24.5 | 24.0 | 23.6 | 23.2 | 22.7 | 22.3 | 21.9 | 21.4 | 21 |
| (between -2 et -3 sd) | 13.5 | 12.5 | 11.5 | 10.5 | 9.6 | 8.6 | 7.6 | 6.6 | 5.6 | 4.6 |
| (less than -3 sd) | 6.0 | 5.4 | 4.7 | 4.1 | 3.5 | 2.9 | 2.3 | 1.6 | 1.0 | 0.4 |
| 12-23 months |  |  |  |  |  |  |  |  |  |  |
| (less than -1 sd | 58.4 | 60.2 | 61.9 | 63.6 | 65.4 | 67.1 | 68.8 | 70.5 | 72.3 | 74 |
| (between -1 et -2 sd) | 23.1 | 22.9 | 22.7 | 22.4 | 22.2 | 21.9 | 21.7 | 21.5 | 21.2 | 21 |
| (between -2 et -3 sd) | 11.7 | 10.9 | 10.1 | 9.3 | 8.5 | 7.8 | 7.0 | 6.2 | 5.4 | 4.6 |
| (less than -3 sd) | 6.7 | 6.0 | 5.3 | 4.6 | 3.9 | 3.2 | 2.5 | 1.8 | 1.1 | 0.4 |
| 24-59 months |  |  |  |  |  |  |  |  |  |  |
| (less than -1 sd | 73.3 | 73.4 | 73.5 | 73.5 | 73.6 | 73.7 | 73.8 | 73.8 | 73.9 | 74 |
| (between -1 et -2 sd) | 17.5 | 17.9 | 18.3 | 18.6 | 19.0 | 19.4 | 19.8 | 20.2 | 20.6 | 21 |
| (between -2 et -3 sd) | 5.5 | 5.4 | 5.3 | 5.2 | 5.1 | 5.0 | 4.9 | 4.8 | 4.7 | 4.6 |
| (less than -3 sd) | 3.7 | 3.4 | 3.0 | 2.6 | 2.3 | 1.9 | 1.5 | 1.1 | 0.8 | 0.4 |

* 2014 baseline values are estimated based on 2012-13 DHS data, assuming no change from 2012-13 to 2014

**Table 3.** PRODESS/PDDSS interventions and coverage from 2014 to 2023

|  | **Years of decennial plan implementation** | | | | | | | | | |
| --- | --- | --- | --- | --- | --- | --- | --- | --- | --- | --- |
| **Interventions** | *2014*  *baseline* | *2015* | *2016* | *2017* | *2018* | *2019* | *2020* | *2021* | *2022* | *2023*  *Endline* |
| **Periconceptual** |  |  |  |  |  |  |  |  |  |  |
| Prevalence contraceptive | 11 | 11.7 | 12.7 | 13.7 | 14.8 | 15.8 | 16.9 | 17.9 | 19.0 | 20 |
| **Pregnancy** |  |  |  |  |  |  |  |  |  |  |
| Antenatal care | 41 | 43.9 | 46.5 | 49.2 | 51.8 | 54.4 | 57.1 | 59.7 | 62.4 | 65 |
| Intermittent Preventive  Treatment of malaria  during pregnancy (IPTp) | 73 | 75.1 | 76.9 | 78.8 | 80.7 | 82.5 | 84.4 | 86.3 | 88.1 | 90 |
| **Childbirth** |  |  |  |  |  |  |  |  |  |  |
| Skilled birth attendance | 60 | 63.3 | 66.7 | 70.0 | 73.3 | 76.7 | 80.0 | 83.3 | 86.7 | 90 |
| Health facility delivery | 60 | 63.3 | 66.7 | 70.0 | 73.3 | 76.7 | 80.0 | 83.3 | 86.7 | 90 |
| Unassisted delivery | 40 | 36.7 | 33.3 | 30.0 | 26.7 | 23.3 | 20.0 | 16.7 | 13.3 | 10 |
| BEmOC | 9 | 11.3 | 13.7 | 16.0 | 18.3 | 20.7 | 23.0 | 25.3 | 27.7 | 30 |
| **Stunting** | 37 | 34.8 | 32.3 | 29.8 | 27.4 | 24.9 | 22.4 | 19.9 | 17.5 | 15 |
| <1 month |  |  |  |  |  |  |  |  |  |  |
| (less than -1 sd | 68.7 | 68.7 | 68.7 | 68.7 | 68.7 | 68.7 | 68.7 | 68.7 | 68.7 | 68.7 |
| (between -1 et -2 sd) | 16.1 | 16.1 | 16.1 | 16.2 | 16.2 | 16.2 | 16.2 | 16.3 | 16.3 | 16.3 |
| (between -2 et -3 sd) | 10.2 | 10.5 | 10.8 | 11.1 | 11.4 | 11.7 | 12.0 | 12.3 | 12.6 | 12.9 |
| (less than -3 sd) | 5.0 | 4.7 | 4.3 | 4.0 | 3.7 | 3.4 | 3.0 | 2.7 | 2.4 | 2.1 |
| 1-5 months |  |  |  |  |  |  |  |  |  |  |
| (less than -1 sd | 68.7 | 68.7 | 68.7 | 68.7 | 68.7 | 68.7 | 68.7 | 68.7 | 68.7 | 68.7 |
| (between -1 et -2 sd) | 16.1 | 16.1 | 16.1 | 16.2 | 16.2 | 16.2 | 16.2 | 16.3 | 16.3 | 16.3 |
| (between -2 et -3 sd) | 10.2 | 10.5 | 10.8 | 11.1 | 11.4 | 11.7 | 12.0 | 12.3 | 12.6 | 12.9 |
| (less than -3 sd) | 5.0 | 4.7 | 4.3 | 4.0 | 3.7 | 3.4 | 3.0 | 2.7 | 2.4 | 2.1 |
| 6-11 months |  |  |  |  |  |  |  |  |  |  |
| (less than -1 sd | 62.9 | 62.9 | 62.9 | 62.9 | 62.9 | 63.0 | 63.0 | 63.0 | 63.0 | 63.0 |
| (between -1 et -2 sd) | 20.3 | 20.5 | 20.7 | 20.9 | 21.0 | 21.2 | 21.4 | 21.6 | 21.8 | 22.0 |
| (between -2 et -3 sd) | 9.0 | 9.5 | 9.9 | 10.3 | 10.7 | 11.2 | 11.6 | 12.0 | 12.5 | 12.9 |
| (less than -3 sd) | 7.8 | 7.2 | 6.5 | 5.9 | 5.3 | 4.6 | 4.0 | 3.4 | 2.7 | 2.1 |
| 12-23 months |  |  |  |  |  |  |  |  |  |  |
| (less than -1 sd | 37.1 | 38.6 | 40.2 | 41.8 | 43.4 | 45.0 | 46.6 | 48.2 | 49.8 | 51.4 |
| (between -1 et -2 sd) | 25.1 | 26.0 | 27.0 | 27.9 | 28.9 | 29.8 | 30.8 | 31.7 | 32.7 | 33.6 |
| (between -2 et -3 sd) | 19.3 | 18.5 | 17.8 | 17.1 | 16.4 | 15.7 | 15.0 | 14.3 | 13.6 | 12.9 |
| (less than -3 sd) | 18.7 | 16.8 | 15.0 | 13.1 | 11.3 | 9.5 | 7.6 | 5.8 | 3.9 | 2.1 |
| 24-59 months |  |  |  |  |  |  |  |  |  |  |
| (less than -1 sd | 32.1 | 34.2 | 36.4 | 38.5 | 40.7 | 42.8 | 45.0 | 47.1 | 49.3 | 51.4 |
| (between -1 et -2 sd) | 22.9 | 24.1 | 25.2 | 26.4 | 27.6 | 28.8 | 30.0 | 31.2 | 32.4 | 33.6 |
| (between -2 et -3 sd) | 22.1 | 21.1 | 20.1 | 19.0 | 18.0 | 17.0 | 16.0 | 14.9 | 13.9 | 12.9 |
| (less than -3 sd) | 23.0 | 20.7 | 18.3 | 16.0 | 13.7 | 11.4 | 9.1 | 6.7 | 4.4 | 2.1 |
| **Wasting** | 13 | 12.1 | 11.2 | 10.3 | 9.4 | 8.5 | 7.7 | 6.8 | 5.9 | 5 |
| <1 month |  |  |  |  |  |  |  |  |  |  |
| (less than -1 sd | 60.5 | 62.0 | 63.5 | 65.0 | 66.5 | 68.0 | 69.5 | 71.0 | 72.5 | 74 |
| (between -1 et -2 sd) | 21.5 | 21.4 | 21.4 | 21.3 | 21.3 | 21.2 | 21.2 | 21.1 | 21.1 | 21 |
| (between -2 et -3 sd) | 7.4 | 7.1 | 6.8 | 6.5 | 6.2 | 5.9 | 5.5 | 5.2 | 4.9 | 4.6 |
| (less than -3 sd) | 10.6 | 9.5 | 8.4 | 7.2 | 6.1 | 4.9 | 3.8 | 2.7 | 1.5 | 0.4 |
| 1-5 months |  |  |  |  |  |  |  |  |  |  |
| (less than -1 sd | 60.5 | 62.0 | 63.5 | 65.0 | 66.5 | 68.0 | 69.5 | 71.0 | 72.5 | 74 |
| (between -1 et -2 sd) | 21.5 | 21.4 | 21.4 | 21.3 | 21.3 | 21.2 | 21.2 | 21.1 | 21.1 | 21 |
| (between -2 et -3 sd) | 7.4 | 7.1 | 6.8 | 6.5 | 6.2 | 5.9 | 5.5 | 5.2 | 4.9 | 4.6 |
| (less than -3 sd) | 10.6 | 9.5 | 8.4 | 7.2 | 6.1 | 4.9 | 3.8 | 2.7 | 1.5 | 0.4 |
| 6-11 months |  |  |  |  |  |  |  |  |  |  |
| (less than -1 sd | 55.6 | 57.6 | 59.7 | 61.7 | 63.8 | 65.8 | 67.9 | 69.9 | 72.0 | 74 |
| (between -1 et -2 sd) | 24.9 | 24.5 | 24.0 | 23.6 | 23.2 | 22.7 | 22.3 | 21.9 | 21.4 | 21 |
| (between -2 et -3 sd) | 13.5 | 12.5 | 11.5 | 10.5 | 9.6 | 8.6 | 7.6 | 6.6 | 5.6 | 4.6 |
| (less than -3 sd) | 6.0 | 5.4 | 4.7 | 4.1 | 3.5 | 2.9 | 2.3 | 1.6 | 1.0 | 0.4 |
| 12-23 months |  |  |  |  |  |  |  |  |  |  |
| (less than -1 sd | 58.4 | 60.2 | 61.9 | 63.6 | 65.4 | 67.1 | 68.8 | 70.5 | 72.3 | 74 |
| (between -1 et -2 sd) | 23.1 | 22.9 | 22.7 | 22.4 | 22.2 | 21.9 | 21.7 | 21.5 | 21.2 | 21 |
| (between -2 et -3 sd) | 11.7 | 10.9 | 10.1 | 9.3 | 8.5 | 7.8 | 7.0 | 6.2 | 5.4 | 4.6 |
| (less than -3 sd) | 6.7 | 6.0 | 5.3 | 4.6 | 3.9 | 3.2 | 2.5 | 1.8 | 1.1 | 0.4 |
| 24-59 months |  |  |  |  |  |  |  |  |  |  |
| (less than -1 sd | 73.3 | 73.4 | 73.5 | 73.5 | 73.6 | 73.7 | 73.8 | 73.8 | 73.9 | 74 |
| (between -1 et -2 sd) | 17.5 | 17.9 | 18.3 | 18.6 | 19.0 | 19.4 | 19.8 | 20.2 | 20.6 | 21 |
| (between -2 et -3 sd) | 5.5 | 5.4 | 5.3 | 5.2 | 5.1 | 5.0 | 4.9 | 4.8 | 4.7 | 4.6 |
| (less than -3 sd) | 3.7 | 3.4 | 3.0 | 2.6 | 2.3 | 1.9 | 1.5 | 1.1 | 0.8 | 0.4 |
| **Vaccine** |  |  |  |  |  |  |  |  |  |  |
| DPT - Three doses | 74 | 76.7 | 79.3 | 82.0 | 84.7 | 87.3 | 90.0 | 92.7 | 95.3 | 98 |
| H. influenzae b - Three doses | 74 | 76.7 | 79.3 | 82.0 | 84.7 | 87.3 | 90.0 | 92.7 | 95.3 | 98 |
| HepB - Three doses | 74 | 76.7 | 79.3 | 82.0 | 84.7 | 87.3 | 90.0 | 92.7 | 95.3 | 98 |
| Measles - Single dose | 72 | 75.0 | 77.9 | 80.7 | 83.6 | 86.5 | 89.4 | 92.2 | 95.1 | 98 |
| **WASH** |  |  |  |  |  |  |  |  |  |  |
| Improved sanitation | 24 | 24.5 | 25.2 | 25.9 | 26.6 | 27.2 | 27.9 | 28.6 | 29.3 | 30 |
