# Supplementary material for: Using the Lives Saved Tool to aid country planning in meeting mortality targets: a case study from Mali
Source: BMC Public Health. 2017 Nov 7;17(Suppl 4):777. doi: 10.1186/s12889-017-4749-y (PMC5688424; doi:10.1186/s12889-017-4749-y)
Supplement: Supplementary file 2 — Results from LiST, Word Document. (DOCX 17 kb) [file 12889_2017_4749_MOESM2_ESM.docx]

# Additional file 2- Results from LiST

**Table 1.** Number of newborn lives saved by intervention, from 2014 to 2023 in Mali

| Interventions | Number of live saved by intervention | | |
| --- | --- | --- | --- |
|  | PRODESS/PDDSS | Projection 1 | Projection 2 |
| Labor and delivery management | 17,303 | 17,303 | 17,801 |
| Changes in breastfeeding | 0 | 17,202 | 17,148 |
| Full supportive care for sepsis/pneumonia | 0 | 9,168 | 9,223 |
| Clean birth practices | 3,753 | 3,594 | 3,742 |
| Neonatal resuscitation | 3,299 | 3,299 | 3,486 |
| Immediate assessment and stimulation | 2,726 | 2,726 | 2,864 |
| Thermal care | 0 | 2,633 | 2,625 |
| Clean postnatal practices | 0 | 2,087 | 2,078 |
| Antibiotics for pPRoM | 1,756 | 1,759 | 1,928 |
| TT - Tetanus toxoid vaccination | 0 | 1,657 | 1,657 |
| IPTp/ITN use in Pregnant women | 930 | 930 | 930 |
| Oral antibiotics | 0 | 1,316 | 816 |
| ORS - oral rehydration solution | 0 | 149 | 262 |
| Syphilis detection and treatment | 214 | 124 | 80 |
| ***Total*** | ***29,981*** | ***63,947*** | ***64,640*** |

**Table 2.** Number of maternal lives saved by intervention, from 2014 to 2023 in Mali

| Interventions | Number of live saved by intervention | | |
| --- | --- | --- | --- |
|  | PRODESS/PDDSS | Projection 1 | Projection 2 |
| Labor and delivery management | 1,462 | 1,463 | 1,539 |
| AMTSL | 731 | 731 | 794 |
| MgSO4 management of eclampsia | 660 | 661 | 725 |
| Clean birth practices | 393 | 393 | 408 |
| Antibiotics for pPRoM | 210 | 210 | 231 |
| IPTp/ITN use in Pregnant women | 47 | 47 | 47 |
| TT - Tetanus toxoid vaccination | 0 | 12 | 12 |
| MgSO4 - Management of pre-eclampsia | 18 | 10 | 6 |
| Hypertensive disorder case management | 15 | 9 | 5 |
| Calcium supplementation | 0 | 3 | 0 |
| ***Total*** | ***3,536*** | ***3,539*** | ***3,767*** |

**Table 3.** Number of under-five lives saved by intervention, from 2014 to 2023 in Mali

| Interventions | Number of live saved by intervention | | |
| --- | --- | --- | --- |
|  | PRODESS/PDDSS | Projection 1 | Projection 2 |
| Antimalarials - Artemesinin compounds for malaria | 0 | 27,640 | 38,719 |
| Stunting | 37,489 | 45,885 | 35,082 |
| Wasting | 39,742 | 34,234 | 31,487 |
| Changes in breastfeeding | 0 | 27,199 | 27,222 |
| Labor and delivery management | 17,303 | 17,303 | 17,801 |
| ITN/IRS | 0 | 11,334 | 11,338 |
| H. influenzae b | 10,215 | 9,812 | 9,996 |
| ORS - oral rehydration solution | 0 | 5,156 | 9,363 |
| Full supportive care for sepsis/pneumonia | 0 | 9,168 | 9,223 |
| Hand washing with soap | 0 | 8,352 | 8,331 |
| Pneumococcal | 2,607 | 7,871 | 7,793 |
| Oral antibiotics for pneumonia | 0 | 6,885 | 6,594 |
| Clean birth practices | 3,753 | 3,594 | 3,742 |
| Neonatal resuscitation | 3,299 | 3,299 | 3,486 |
| Immediate assessment and stimulation | 2,726 | 2,726 | 2,864 |
| Thermal care | 0 | 2,633 | 2,625 |
| Clean postnatal practices | 0 | 2,087 | 2,078 |
| Rotavirus | 0 | 1,032 | 2,057 |
| Antibiotics for pPRoM | 1,756 | 1,759 | 1,928 |
| DPT | 1,765 | 1,780 | 1,781 |
| TT - Tetanus toxoid vaccination | 0 | 1,657 | 1,657 |
| Measles | 1,205 | 1,167 | 1,260 |
| Zinc - for treatment of diarrhea | 0 | 1,841 | 1,218 |
| Improved sanitation - Utilization of latrines or toilets | 1,354 | 1,181 | 1,177 |
| Improved water source | 0 | 1,129 | 1,127 |
| Vitamin A supplementation | 0 | 505 | 1,052 |
| IPTp/ITN use in Pregnant women | 930 | 930 | 930 |
| Oral antibiotics | 0 | 1,316 | 816 |
| Antibiotics - for treatment of dysentery | 0 | 235 | 576 |
| Water connection in the home | 0 | 441 | 440 |
| ART | 311 | 312 | 312 |
| Zinc supplementation | 0 | 250 | 251 |
| Vitamin A - for treatment of measles | 0 | 115 | 121 |
| Syphilis detection and treatment | 214 | 124 | 80 |
| ***Total*** | ***124,669*** | ***240,952*** | ***244,527*** |
